# Longitudinal reciprocal associations between volunteering, health and well-being: evidence for middle-aged and older adults in Europe

**DOI:** 10.1093/eurpub/ckae014

**Published:** 2024-02-22

**Authors:** Dorota Weziak-Bialowolska, Regina Skiba, Piotr Bialowolski

**Affiliations:** Centre for Evaluation and Analysis of Public Policies, Faculty of Philosophy, Jagiellonian University, Cracow, Poland; Department of Quantitative Methods & Information Technology, Kozminski University, Warsaw, Poland; Human Flourishing Program, Institute for Quantitative Social Science, Harvard University, Cambridge, MA, USA; Centre for Evaluation and Analysis of Public Policies, Faculty of Philosophy, Jagiellonian University, Cracow, Poland; Human Flourishing Program, Institute for Quantitative Social Science, Harvard University, Cambridge, MA, USA; Department of Economics, Kozminski University, Warsaw, Poland

## Abstract

**Background:**

There is substantial evidence on the importance of voluntary activities for the health of middle-aged and older adults. Evidence on the effects of health and well-being on volunteering is more limited. This study examines reciprocal longitudinal associations between voluntary and/or charity activities and 21 indicators related to physical health, well-being, cognitive impairment and daily life functioning among middle-aged and older adults.

**Methods:**

Longitudinal data were collected between 2011 and 2020 from a sample of 19 821 middle-aged and older adults from 15 countries participating in the Survey of Health, Ageing and Retirement in Europe. An outcome-wide analysis and an exposure-wide analysis were applied and generalized estimating equations were used. Robustness analyses were conducted.

**Results:**

Voluntary and/or charity activities almost every week or more often were prospectively associated with greater emotional well-being, reduced risks of limitations in activities of daily living and of being diagnosed with Alzheimer’s disease at a 6-year follow-up. Positive reverse associations were found from emotional well-being to volunteering. Limitations in daily life activities, increased loneliness, high blood cholesterol, hypertension and chronic lung disease were found to impede participation in voluntary and/or charity activities over time. Feedback loops between voluntary and/or charity activities and well-being and limitations in daily activities may exist.

**Conclusion:**

Understanding the reciprocal nature of the relationship between volunteering and health and well-being can help identify strategies to encourage volunteering among middle-aged and older adults with specific health conditions and to target health promotion efforts towards volunteers.

## Introduction

Volunteering is defined as an activity where individuals provide help without receiving payment, with the goal of benefiting others or a cause.^1^ Participating in volunteering can be beneficial not only for the recipients, but also for the volunteers themselves. According to Eurostat, 40% of adult Europeans participate in voluntary activities with prevalence rates of 19.9% among Europeans aged 50–64 and 17.8% among those aged 65 and above. While the frequency of volunteering varies across different European countries, there is evidence that these activities are associated with positive health outcomes, healthy lifestyles and well-being, particularly among middle-aged and older adults.^2^^,^^3^

Prior research on the health effects of volunteering for middle-aged and older adults showed its favorable association with longevity,^4^ survival^5^ and reduced risk of mortality.^6^^,^^7^ Additionally, scholars have reported associations between volunteering and well-being and health among middle-aged and older adults in terms of improved quality of life,^8^ greater well-being including life satisfaction, purpose in life and positive affect,^6^^,^^9–11^ better physical health outcomes (e.g. reduced risk of hypertension and cardiovascular disease^12–14^), reduced physical functioning limitations^6^ and delay in the progression of physical disability,^15^ as well as lower risks of loneliness,^6^ cognitive impairment^16^ and depressive symptoms.^3^^,^^6^ There is also some evidence that volunteering could be detrimental for life satisfaction and hypertension, especially when older adults engage too much in voluntary work.^13^^,^^17^^,^^18^

Some evidence, albeit limited, regarding the reverse association, that is, on the effect of health on frequency of volunteering for middle-aged and older Europeans is also available. Notably, studies have revealed that chronic diseases, mobility limitations and experiences of depression among this demographic are linked to reduced engagement in volunteer activities.^19^^,^^20^

Although prior studies have extensively emphasized the significance of voluntary activities for the health of middle-aged and older adults, they less often explored the reciprocal effects of health on volunteering, despite evidence that such associations could exist.^19^^,^^20^ Therefore, our study focuses on examining the bidirectional longitudinal associations between the frequency of volunteering and/or charity-related activities and various indicators related to physical health, emotional well-being, cognitive impairment and daily life functioning (PH-EWB-CI-DLF) among middle-aged and older adults in Europe. The research questions we address are as follows: (i) What changes in PH-EWB-CI-DLF indicators could be observed within a 6-year time horizon, if middle-aged and older people engage in voluntary and charity-related activities? And (ii) How are PH-EWB-CI-DLF factors associated with subsequent (observed within a 6-year timeframe) changes in voluntary and charity-related activities by middle-aged and older adults?

## Methods

### Study population

The study used longitudinal data from the Survey of Health, Ageing and Retirement in Europe (SHARE).^21^ SHARE is a comprehensive biennial panel study designed to assess health and socio-economic living conditions of individuals aged 50 and above across Europe. Detailed documentation and access to the datasets are available on the SHARE website (https://share-eric.eu). Ethical approval for the study was obtained from the Ethics Committee of the University of Mannheim and the Ethics Council of the Max Planck Society. All participants provided an informed consent to participate in the study.

The analytical sample comprised all respondents who participated in SHARE in waves 4, 5 and 8 (2011–2020) and provided data on the frequency of volunteering, charity activity and PH-EWB-CI-DLF variables. No specific exclusion criteria were applied. In total, data from 19 821 middle-aged and older adults aged 50 years or older were examined. Respondents were from 15 European countries, including Austria, Germany, Sweden, the Netherlands, Spain, Italy, France, Denmark, Switzerland, Belgium, Czechia, Poland, Hungary, Slovenia and Estonia.

### Measures

#### Volunteering

Volunteer activity was assessed by asking respondents about the frequency of doing voluntary or charity work in the last 12 months (almost every day, almost every week, almost every month, less often, never). For the analyses, this variable was recoded into three categories: almost every week or more often, almost every month or less often, never.

#### Emotional well-being

Five single items related to well-being were considered. They were derived from the CASP-12 questionnaire, which measures quality of life in early old age.^22^ These items were as follows: (i) ‘Future looks good’, (ii) ‘I feel full of energy these days’, (iii) ‘On balance, I look back at my life with a sense of happiness’, (iv) ‘I look forward to each day’ and (v) ‘I feel that my life has meaning’. Additionally, a sense of loneliness was measured using a short version of the UCLA Loneliness Scale.^23^ The self-reported diagnosis of Alzheimer’s disease, dementia, or other serious memory impairment was also considered, as well as self-reported depression using the EURO-D geriatric depression scale.^24^^,^^25^

#### Daily life functioning

Two indicators of daily life functioning that measure the level of difficulty due to physical, mental, emotional and memory problems with activities of daily living (ADL) and instrumental activities of daily living (IADL) were used.^26^ The scores range from 0 to 6 or 7, respectively. Values above 0 indicate reported limitations with ADL and/or IADL.

#### Physical health

The following physical health outcomes were examined: heart attack, hypertension, high blood cholesterol, stroke, diabetes, chronic lung disease, and cancer. Furthermore, we considered two additional variables: the presence of impairing pain and mobility limitations.

#### Cognitive impairment

Cognitive impairment was considered using a measure of time orientation (respondents were asked about awareness of the current year, month and day of the month, as well as about day of the week). This summary measure ranges from 0 to 4.^27^

The Supplementary material S1 provides further details about each of the variables described in this section.

#### Covariates

Consistent with previous research, various factors have been identified as predictors of well-being and health. These include demographic characteristics, work, education, lifestyle and health conditions.^28^^,^^29^ Additionally, personality traits have been shown to not only predict a propensity for volunteering, but also moderate the association between volunteerism and health.^30^^,^^31^ Therefore, this study accounted for these important variables.

Specifically, we controlled for demographic characteristics (gender, age, marital status, educational attainment, employment status and country), socioeconomic factors (annual personal income and net financial assets of households), personality traits (measured using the 10-item Big Five Inventory [BFI-10]), health behaviors (sports activity requiring moderate effort, alcohol consumption and BMI), and health history; all self-reported and measured in the pre-baseline wave, that is, wave 4, as they could confound the examined associations.

The Supplementary material S1 provides further details about each covariate variable described in this section.

#### Prior values of PH-EWB-CI-DLF variables and volunteering

Given evidence indicating that health status might affect the subsequent decision to volunteer and conversely, volunteering activities may affect subsequent health and well-being outcomes,^11^^,^^28^^,^^29^ to reduce the possibility of reverse causation and residual confounding, in each model we adjusted for the pre-baseline values of the 21 PH-EWB-CI-DLF variables (in an outcome-wide analysis—simultaneously) and for pre-baseline values of volunteering (in an exposure-wide analysis).

## Statistical analysis

An outcome-wide analysis was applied to examine the prospective associations between volunteering and the subsequent 21 PH-EWB-CI-DLF variables (previously examined in individual studies).^32^ An exposure-wide analysis was applied to investigate the prospective associations between 21 PH-EWB-CI-DLF variables and subsequent volunteering. The aim was to extensively examine the pattern of reciprocal associations between volunteering and the PH-EWB-CI-DLF variables. This approach offers certain advantages, including uncovering patterns of associations that might otherwise remain unnoticed when examining a single outcome/exposure. It also serves as a safeguard against selectively presenting only significant results, thereby reducing the risk of cherry-picking findings.

This study used data from three time points (wave 4 ≈ 2011, wave 5 ≈ 2013, wave 8 ≈ 2019–2020). The covariates were evaluated in the pre-baseline wave (wave 4) so that they are confounding and not intervening variables, with the exception of personality traits that were assessed only once in SHARE, in wave 7. Despite the potential for personality traits to develop over the life course, evidence suggests their relative stability over shorter periods.^33^ The independent variable was assessed in the baseline wave (wave 5) and the dependent variables were assessed 6 years later in the outcome wave (wave 8).

Reciprocal longitudinal associations were examined using generalized estimating equations. The reported estimates included: (i) standardized regression estimates (for continuous variables); (ii) odds ratios (for dichotomous and rare variables occurring in less than 10% of the population as well as for ordered variables); (iii) risk ratios (for dichotomous and non-rare variables occurring in at least 10% of the population). Risk ratios were estimated using a modified Poisson regression with robust standard errors.^34^ Adjustment for standard errors and clustering by country were applied to address the nested nature of the data. The significance level after Bonferroni correction for multiple testing was reported. However, given the ongoing discussion on applicability of measures designed to make such a correction,^35^ we interpreted the results based on the uncorrected significance levels. Missing variables were imputed using chained equations (10 sets of imputed data were generated^36^) and multiple imputation estimates were pooled using Rubin’s rule.^37^

A series of robustness checks was conducted. First, the robustness of the results against unmeasured confounding was examined using *E*-values.^38^ This sensitivity measure assesses the magnitude to which an unaccounted confounder would need to be associated with both the exposure and the outcome to explain the observed association. Second, to address potential feedback loops, the prospective associations between volunteering and/or charity activity and each of the PH-EWB-CI-DLF indicators, and vice versa, were examined simultaneously using the cross-lagged panel model.^39^ This analysis was performed in two ways: (i) treating the volunteering and/or charity variable as ordinal variable as in primary analysis, and (ii) treating volunteering variable as continuous variable (Supplementary tables S2 and S3). Third, the primary models were reanalyzed using a complete case scenario to assess the robustness of the results against missing data patterns (Supplementary table S4). Next, the primary models were rerun with limited sets of controls (Supplementary table S5) to examine the risk of overfitting. Model 1 controlled for gender and age, while Model 2, in addition to gender and age, included employment status and wealth. Both models controlled for the prior outcomes of interest. Model 3 adjusted for all covariates as in the primary model but only considered the prior outcome of interest, compared with simultaneous control for all outcomes in the primary models. Finally, the primary models were also rerun with the volunteering variable treated as a continuous one (Supplementary table S6).

All statistical analyses were performed using Stata/SE 17.0.

## Results

In the pre-baseline wave, participants had an average age of 64.5 years (SD = 8.72), were mostly women and married, with upper secondary or first-stage tertiary education. More than 10% of the participants reported doing voluntary and/or charity work almost every week or more often, 9.0% —almost every month or less often, and 80.6% never (detailed statistics in table 1 and Supplementary table S1).

**Table 1:** Distribution of participant characteristics at study pre-baseline wave (wave 4, *N* = 19 821). Survey of Health, Ageing and Retirement in Europe (SHARE), middle-aged and older adults aged 50 and over

Participant characteristic	Total (*N* = 19 821)^a^		Never (*N* = 15 879^a^)		Almost every month or less often (*N* = 1780^a^)		Almost every week or more often (*N* = 2042^a^)	
	%	Mean (SD)	%	Mean (SD)	%	Mean (SD)	%	Mean (SD)
Sociodemographic factors								
Gender								
Male	40.71		40.08		44.72		41.58	
Female	59.29		59.92		55.28		58.42	
Age group								
50–59	31.06		31.11		37.51		25.12	
60–69	40.07		39.26		40.81		45.98	
70–79	23.36		23.67		19.07		24.58	
80+	5.52		5.96		2.62		4.32	
Marital status								
Married and living together with the spouse	69.61		69.29		72.30		70.24	
Registered partnership	1.48		1.47		1.51		1.43	
Married, but living separately	1.32		1.38		0.87		1.18	
Never married	5.53		5.52		5.28		5.58	
Divorced	9.65		9.46		10.57		10.45	
Widowed	12.41		12.87		9.47		11.12	
Employment status								
Retired	53.76		53.93		46.29		58.90	
Employed or self-employed	31.22		30.98		40.67		25.06	
Unemployed	3.17		3.28		2.81		2.60	
Permanently sick of disabled	3.24		3.43		1.85		2.80	
Homemaker	7.56		7.53		6.97		8.44	
Other	1.05		0.86		1.40		2.21	
Education attainment (ISCED-97)								
None	2.27		2.62		0.56		0.78	
Primary education or first stage of basic education	15.04		16.70		7.42		8.62	
Lower secondary or second stage of basic education	17.95		19.04		12.47		14.15	
(Upper) Secondary education	36.13		36.12		37.58		35.36	
Post-secondary non-tertiary education	5.55		5.46		6.63		5.34	
First stage of tertiary education	22.22		19.42		33.26		34.38	
Second stage of tertiary education	0.84		0.64		2.08		1.37	
Annual personal income (Euro)		32 042 (48 026)		28 626 (44 547)		44 755 (51 107)		47 861 (64 387)
Household net financial assets (Euro)		60 746 (192 095)		51 179 (189 150)		94 246 (199 953)		105 578 (199 356)
Country								
Austria	7.16		6.96		9.49		6.42	
Germany	3.28		3.05		3.37		4.85	
Sweden	4.32		4.48		3.71		3.77	
The Netherlands	5.30		3.73		8.65		14.74	
Spain	6.04		7.04		2.02		1.71	
Italy	5.71		6.37		2.70		3.48	
France	10.72		9.70		13.31		16.50	
Denmark	5.29		4.45		9.16		8.67	
Switzerland	9.05		7.48		15.45		15.92	
Belgium	6.89		5.81		9.72		12.10	
Czech Republic	10.15		11.45		6.40		2.79	
Poland	3.13		3.82		0.56		0.24	
Hungary	3.74		4.14		2.87		1.42	
Slovenia	5.84		6.22		5.00		3.77	
Estonia	13.38		15.32		7.58		3.62	
Personality traits								
Extraversion; 1–5		3.51 (0.94)		3.48 (0.94)		3.64 (0.91)		3.68 (0.91)
Agreeableness; 1–5		3.70 (0.80)		3.68 (0.80)		3.76 (0.78)		3.80 (0.77)
Consciousness; 1–5		4.11 (0.79)		4.09 (0.81)		4.20 (0.72)		4.25 (0.71)
Neuroticism; 1–5		2.58 (1.01)		2.60 (1.01)		2.50 (0.97)		2.49 (1.00)
Openness; 1–5		3.37 (0.97)		3.34 (0.97)		3.58 (0.95)		3.51 (0.95)
Lifestyle factors								
BMI		27.03 (4.75)		27.18 (4.80)		26.64 (4.68)		26.15 (4.29)
Alcohol consumption								
Almost every day	17.44		16.28		21.18		23.21	
5–6 days a week	2.99		2.80		3.37		4.11	
3–4 days a week	7.45		6.77		10.67		9.99	
Once or twice a week	19.57		18.62		23.99		23.16	
Once or twice a month	13.12		12.79		15.11		13.81	
Less than once a month	11.74		12.27		10.34		8.96	
Not at all in the last 6 months	27.69		30.47		15.34		16.75	
Sport activity requiring a moderate level of energy								
More than once a week	73.24		71.35		80.11		73.29	
Once a week	13.38		13.77		12.58		10.92	
One to three times a month	5.39		5.84		4.10		3.09	
Hardly ever or never	7.99		9.04		3.20		3.62	
Outcomes								
Emotional well-being								
Loneliness (3-item loneliness scale; 3–9)		3.70 (1.21)		3.75 (1.25)		3.59 (1.09)		3.51 (1.01)
Alzheimer’s disease	0.51		0.52		0.45		0.15	
Depression (EURO-D ≥ 4)	25.28		26.68		20.00		19.13	
Future looks good (1–4)		3.12 (0.90)		3.04 (0.92)		3.40 (0.76)		3.44 (0.76)
I feel full of energy these days (1–4)		3.26 (0.82)		3.20 (0.84)		3.48 (0.69)		3.53 (0.67)
On balance, I look back at my life with a sense of happiness (1–4)		3.39 (0.77)		3.35 (0.78)		3.55 (0.67)		3.57 (0.68)
I look forward to each day (1–4)		3.49 (0.83)		3.45 (0.84)		3.64 (0.76)		3.65 (0.76)
I feel that my life has meaning (1–4)		3.61 (0.69)		3.57 (0.72)		3.80 (0.54)		3.81 (0.50)
Daily life functioning								
ADL (at least 1 limitation)	7.60		8.10		5.90		4.61	
IADL (at least 1 limitation)	12.31		13.37		7.70		7.01	
Physical health								
Heart attack	10.98		11.54		8.31		8.62	
Hypertension	38.49		39.94		35.06		30.51	
High blood cholesterol	22.55		22.82		20.62		21.99	
Stroke	2.82		2.92		2.02		2.50	
Diabetes	10.59		11.25		8.65		7.15	
Cancer	4.12		4.20		3.54		4.06	
Chronic lung disease	5.60		5.78		4.72		5.00	
Pain	42.55		44.37		36.56		34.88	
Mobility (at least 1 limitation)	45.66		47.55		36.18		38.46	
Cognitive Impairment								
Date orientation; 0–4		3.85 (0.52)		3.86 (0.50)		3.87 (0.45)		3.90 (0.31)

Note: SD, standard deviation; BMI, body mass index.

a Total observation *N* = 19 821, missing data = 820.

Over the 6-year follow-up period, middle-aged and older Europeans who participated in voluntary and/or charity activities almost every week or more frequently had substantially lower odds of being diagnosed with Alzheimer’s disease (*P* = 0.006) (table 2, left panel). Compared with non-participants, they also reported higher scores in three emotional well-being indicators: ‘Future looks good’ (*P* = 0.010), ‘I feel full of energy these days’ (*P* < 0.001), and ‘On balance, I look back on my life with a sense of happiness’ (*P* = 0.003). Additionally, they had a substantially lower risk of limitations in ADL (*P* < 0.001) and in IADL (*P* < 0.001). These prospective associations were independent of demographic and socioeconomic status, personality, prior health history, prior quality of life, health behaviors, lifestyle, and prior involvement in voluntary and/or charity work. There was no evidence supporting a prospective association between participation in voluntary and/or charitable activities and experiences of loneliness, depression or various physical health outcomes, including heart attack, hypertension, stroke, diabetes, chronic lung disease, cancer, pain, mobility limitations and cognitive impairment at the 6-year follow-up.

**Table 2:** Prospective reciprocal associations between voluntary and/or charity work and emotional well-being, physical health, daily life functioning and cognitive impairment; Survey of Health, Ageing and Retirement in Europe (SHARE), adults aged 50 and over (*N* = 19 821), unidirectional associations

Outcome	Voluntary and/or charity activity (ref. = never) → subsequent PH-EWB-CI-DLF outcome			PH-EWB-CI-DLF indicator → subsequent voluntary and/or charity activity	
	Statistic	Almost every month or less often	Almost every week or more often	Statistic	Ordinal logit model estimate
Emotional well-being					
Loneliness (three-item loneliness scale)	β^a^	−0.023	−0.019	OR	0.939
	(95% CI)	(−0.070; 0.025)	(−0.061; 0.023)	(95% CI)	(0.892; 0.989)
	*P*-value	0.316	0.341	*P*-value	0.017
Alzheimer’s disease	OR	0.639	0.543	OR	1.099
	(95% CI)	(0.364; 1.121)	(0.351; 0.838)	(95% CI)	(0.536; 1.253)
	*P*-value	0.118	0.006	*P*-value	0.796
Depression (EURO-D ≥ 4)	RR	0.987	1.033	OR	0.860
	(95% CI)	(0.907; 1.074)	(0.953; 1.119)	(95% CI)	(0.727; 1.018)
	*P*-value	0.762	0.435	*P*-value)	0.079
Future looks good	β^a^	0.052	0.070	OR	1.083
	(95% CI)	(0.005; 0.100)	(0.020; 0.121)	(95% CI)	(1.024; 1.145)
	*P*-value	0.034	0.010	*P*-value	0.005
I feel full of energy these days	β^a^	0.046	0.114	OR	1.149
	(95% CI)	(−0.034; 0.126)	(0.065; 0.164)	(95% CI)	(1.093; 1.210)
	*P*-value	0.226	<.001	*P*-value	<.001
On balance, I look back on my life with a sense of happiness	β^a^	0.022	0.074	OR	1.007
	(95% CI)	(−0.028; 0.072)	(0.031; 0.117)	(95% CI)	(0.962; 1.055)
	*P*-value	0.349	0.003	*P*-value	0.758
I look forward to each day	β^a^	0.022	0.039	OR	0.977
	(95% CI)	(−0.028; 0.071)	(−0.010; 0.088)	(95% CI)	(0.942; 1.016)
	*P*-value	0.344	0.109	*P*-value	0.217
I feel that my life has meaning	β^a^	0.040	0.053	OR	1.078
	(95% CI)	(−0.012; 0.092)	(−0.007; 0.112)	(95% CI)	(1.026; 1.133)
	*P*-value	0.117	0.077	*P*-value	0.003
Daily life functioning					
ADL (at least 1 limitation)	OR	0.791	0.683	OR	0.737
	(95% CI)	(0.645; 0.970)	(0.575; 0. 812)	(95% CI)	(0.569; 0.953)
	*P*-value	0.024	<.001	*P*-value	0.020
IADL (at least 1 limitation)	RR	0.833	0.826	OR	0.811
	(95% CI)	(0.745; 0.930)	(0.749; 0.911)	(95% CI)	(0.661; 0.993)
	*P*-value	0.001	<.001	*P*-value	0. 043
Physical health					
Heart attack	RR	0.865	0.950	OR	1.010
	(95% CI)	(0.689; 1.087)	(0.837; 1.079)	(95% CI)	(0.860; 1.182)
	*P*-value	0.214	0.432	*P*-value	0.905
Hypertension	RR	1.005	1.010	OR	0.897
	(95% CI)	(0.965; 1.046)	(0.936; 1.089)	(95% CI)	(0.807; 0.996)
	*P*-value	0.813	0.806	*P*-value	0.042
High blood cholesterol	RR	1.064	1.006	OR	0.829
	(95% CI)	(1.004; 1.128)	(0.899; 1.126)	(95% CI)	(0.721; 0.953)
	*P*-value	0.037	0.918	*P*-value	0.008
Stroke	OR	0.644	0.910	OR	0.812
	(95% CI)	(0.413; 1.004)	(0.690; 1.199)	(95% CI)	(0.620; 1.063)
	*P*-value	0.053	0.499	*P*-value	0.129
Diabetes	RR	0.978	0.916	OR	0.995
	(95% CI)	(0.864; 1.107)	(0.829; 1.011)	(95% CI)	(0.798; 1.241)
	*P*-value	0.729	0.080	*P*-value	0.966
Cancer	OR	1.017	0.934	OR	1.114
	(95% CI)	(0.801; 1.292)	(0.748; 1.165)	(95% CI)	(0.882; 1.406)
	*P*-value	0.888	0.543	*P*-value	0.364
Chronic lung disease	OR	0.965	1.234	OR	0.640
	(95% CI)	(0.724; 1.286)	(0.990; 1.539)	(95% CI)	(0.477; 0.858)
	*P*-value	0.807	0.062	*P*-value	0.003
Pain	RR	0.963	0.983	OR	0.906
	(95% CI)	(0.900; 1.030)	(0.926; 1.044)	(95% CI)	(0.821; 1.001)
	*P*-value	0.273	0.581	*P*-value	0.051
Mobility (at least 1 limitation)	RR	0.997	0.997	OR	0.904
	(95% CI)	(0.942; 1.056)	(0.954; 1.042)	(95% CI)	(0.809; 1.011)
	*P*-value	0.930	0.893	*P*-value	0.077
Cognitive impairment					
Date orientation	β^a^	0.017	0.044	OR	1.021
	(95% CI)	(−0.029; 0.062)	(−0.010; 0.099)	(95% CI)	(0.951; 1.096)
	*P*-value	0.437	0.102	*P*-value	0.570

Note: CI, confidence interval; OR, odds ratio; RR, risk ratio; ADL, activities of daily living; IADL, instrumental activities of daily living. Missing covariate variables were imputed using chained equations (10 sets of imputed data were generated). All models were controlled for participant demographics: age, gender, marital status, educational attainment, labor market status, country; socio-economic factors: annual personal income, household net financial assets; health behaviors such as BMI, alcohol consumption and sports activity; and personality traits: agreeableness, openness, conscientiousness, neuroticism, extraversion. Each model was also adjusted for the prior values of the 21 outcome variables, all simultaneously in each regression model, and for the prior value of the exposure variable. The P values cut-off for Bonferroni correction = 0.05/21 indicators = 0.0024.

a All continuous outcomes were standardized (mean = 0, standard deviation = 1), and *β* was the standardized effect size.

Regarding reciprocal longitudinal associations, that is, between PH-EWB-CI-DLF factors and subsequent volunteering and/or charity activities, the study found that middle-aged and older adults in Europe reporting a higher sense of meaning in life as well as greater self-realization (‘Future looks good’ and ‘I feel full of energy these days’) engaged more frequently in volunteering and/or charity activities (*P* = 0.003; *P* = 0.005; and *P* < 0.001, respectively) (table 2, right panel). However, loneliness, limitations in ADL and IADL as well as hypertension, high blood cholesterol and chronic lung disease hindered subsequent participation in voluntary and/or charity activities (*P* = 0.017; *P* = 0.020; *P* = 0.043; *P* = 0.042; *P* = 0.008; and *P* = 0.003, respectively) (table 2, right panel). Five out of these associations (i.e. limitations in ADL and IADL, high blood cholesterol, ‘Future looks good’ and ‘I feel full of energy these days’) exist in both directions suggesting the possible existence of feedback loops and possibly ripple effects between voluntary and/or charity activities and these variables.

### Robustness analysis

The *E*-values indicated that the observed associations were modestly robust to unmeasured confounding (table 3). The most robust associations, evidenced by the highest *E*-value, were found between voluntary and/or charity activities and subsequent reports of Alzheimer’s disease, as well as limitations with ADL. Concerning reciprocal associations, the most robust was this between chronic lung disease and subsequent volunteering and/or charity activity.

**Table 3:** Robustness to unmeasured confounding (*E*-values) for assessing prospective reciprocal associations between voluntary and/or charity work and emotional well-being, physical health, daily life functioning and cognitive impairment (*N* = 19 821); Survey of Health, Ageing and Retirement in Europe (SHARE), adults aged 50 and over

Outcome	Voluntary and/or charity activity (ref. = never) → subsequent PH-EWB-CI-DLF outcome				PH-EWB-CI-DLF indicator → subsequent voluntary and/or charity activity	
	Almost every week or more often		Almost every month or less often		*E*-value for effect estimate	*E*-value for CI limit
	*E*-value for effect estimate	*E*-value for CI limit	*E*-value for effect estimate	*E*-value for CI limit		
Emotional well-being						
Loneliness (3-item loneliness scale)	1.15	1.00	1.14	1.00	1.33	1.12
Alzheimer’s disease	2.51	1.00	3.09	1.67	1.43	1.00
Depression (EURO-D ≥ 4)	1.13	1.00	1.22	1.00	1.60	1.00
Future looks good	1.29	1.11	1.36	1.19	1.38	1.18
I feel full of energy these days	1.29	1.00	1.53	1.38	1.56	1.41
On balance, I look back on my life with a sense of happiness	1.19	1.00	1.41	1.26	1.09	1.00
I look forward to each day	1.18	1.00	1.26	1.00	1.18	1.00
I feel that my life has meaning	1.35	1.01	1.29	1.00	1.37	1.19
Quality of life						
ADL (at least 1 limitation)	1.84	1.21	2.29	1.77	2.05	1.28
IADL (at least 1 limitation)	1.69	1.36	1.72	1.43	1.77	1.09
Physical health						
Heart attack	1.58	1.00	1.29	1.00	1.11	1.00
Hypertension	1.08	1.00	1.11	1.00	1.47	1.07
High blood cholesterol	1.32	1.07	1.08	1.00	1.71	1.28
Stroke	2.48	1.00	1.43	1.00	1.77	1.00
Diabetes	1.07	1.00	1.41	1.00	1.08	1.00
Cancer	1.15	1.00	1.35	1.00	1.47	1.00
Chronic lung disease	1.23	1.00	1.77	1.00	2.50	1.60
Pain	1.24	1.00	1.15	1.00	1.44	1.00
Mobility (at least 1 limitation)	1.06	1.00	1.06	1.00	1.45	1.00
Cognitive impairment						
Date orientation	1.20	1.00	1.38	1.00	1.17	1.00

Notes: CI, confidence interval; ADL, activities of daily living; IADL, instrumental activities of daily living. An unmeasured confounder would need to be associated with both the risk of Alzheimer’s disease and voluntary and/or charity activities almost every week or more often by risk ratios of 3.09 each, above and beyond the measured covariates, to fully explain away the observed association between voluntary and/or charity activities almost every week or more often and subsequent lower risk of Alzheimer’s disease (at the 6-year follow-up). Regarding the *E*-value for the limit of the 95% CI, this unmeasured confounder would need to be associated with both voluntary and/or charity activities almost every week or more often and subsequent lower risk of Alzheimer’s disease by 1.67-fold each, above and beyond the measured covariates, to change the lower limit of the CI for the observed association between risk of Alzheimer’s disease and voluntary and/or charity activities almost every week or more often to include the null value.

Regarding the supplementary analyses concerning a simultaneous estimation of reciprocal prospective associations using the cross-lagged panel model (Supplementary tables S2 and S3), most associations from the primary analyses were confirmed including the feedback loops and with comparable effect sizes. Some additional associations were also found including the effects of volunteering and charity activity on meaning in life, looking forward to each day, and cognitive impairment, and of depression on volunteering and charity activity. Another feedback loop—between participation in volunteering and charity activities and a sense of meaning in life—was also identified.

When rerunning the models in the complete case scenario, the directionality of most associations was preserved. However, the effects sizes were slightly attenuated and the confidence intervals were wider (Supplementary table S4). Supplementary analyses with the use of limited sets of controls (Supplementary table S5) showed that the pattern of associations changed only slightly with the most pronounced effects observed when the adjustment was for gender, age and the prior outcome only (model 1) with the effects subduing with the addition of additional control variables (model 2). While controlling for the full set of covariates (as in the primary analyses) and only for one prior outcome (the one of interest in a particular analysis), the analyses yielded very similar results to those obtained from the primary analyses (model 3 compared with model 0). Finally, when the models were rerun with the volunteering variable treated as continuous, most associations from the primary analyses were validated for both unidirectional and reciprocal analyses (Supplementary table S6 compared with table 2, and Supplementary table S3 compared with Supplementary table S2). This provided additional evidence supporting the robustness of the temporal bidirectional associations between participation in voluntary and/or charity activities and emotional well-being, physical health, daily life functioning and cognitive impairment.

## Discussion

The results of this study corroborate previously documented findings, emphasizing the favorable associations between volunteering and greater well-being, encompassing life satisfaction, sense of purpose and meaning in life, as well as positive affect.^3^^,^^6^^,^^10^ They also add to prior evidence that these activities conducted for the benefit of others can also contribute to the donor’s happiness, optimism, self-confidence and feeling in control.^40^ Our findings further substantiate the longitudinal associations between volunteer and/or charity work and reduced physical functioning limitations, as reflected in ADL and IADL, as previously reported by Kim et al.^6^

Expanding on earlier evidence, our study sheds light on the influence of health on volunteering. Corresponding to previous research,^20^^,^^29^ we show that physical functioning limitations, increased feelings of loneliness, depression and experiences of pain may hamper engagement in volunteering and/or charity activities among middle-aged and older Europeans. Our work also echoes previous studies indicating that individuals with chronic diseases are less inclined to participate in volunteer activities.^19^ However, our study specifically confirms this association among middle-aged and older adults reporting hypertension, high blood cholesterol, and chronic lung disease. Notably, our research highlights the lack of associations between specific health conditions such as cancer, heart attack, stroke and diabetes and volunteering which is also consistent with prior findings for middle-aged and older Europeans and Americans.^20^^,^^29^ We augment these findings by showing that emotional well-being reflected in positive perception of the future and a sense of current energy (and possibly with a sense of meaning in life), may contribute to more frequent volunteering and charity activities, which, in turn, are associated with subsequent enhanced well-being, suggesting potential feedback loops. Other potential feedback loops may exist between limitations in ADL and IADL and less frequent volunteering. Consequently, this study offers insights for understanding the causal mechanisms underlying the relationship between volunteering and well-being and health, as well as for designing effective interventions that promote both volunteering and well-being. More insights on determinants of volunteering could be helpful for understanding the reciprocal nature of the relationship and consequently lead to the identification of strategies to encourage volunteering among individuals with specific health conditions or to target health promotion efforts towards volunteers.

We did not find evidence supporting prospective associations of volunteering with subsequent loneliness, which contracts with previous findings.^6^ However, the earlier study indicated an effect only when individuals spent at least 100 h/year in volunteering activities; for less frequent engagement (i.e. <50 h/year and 50–99 h/year) no effect was observed. In our study, frequency was measured without specific indication regarding the duration of a singular engagement, thus, it is plausible that we were unable to capture the effect corresponding to the frequency of at least 100 h/year.

Similar to Kim et al.,^6^ our study was unable to corroborate the association between volunteering and a subsequent reduction in the risk of hypertension as reported by other studies.^13^^,^^14^ Notably, these studies did not control for prior volunteerism, while both our study and that by Kim et al.^6^ did include this adjustment. Additionally, Sneed and Cohen^14^ found a significant association with a subsequent reduced risk of hypertension only for highly intensive volunteer activity, that is, at least 200 h/year compared with no such activity, but not for less frequent engagement. This implies that we and Kim et al.^6^ examined how change in volunteering is associated with subsequent outcomes, whereas Burr et al.^13^ and Sneed and Cohen^14^ examined the association between the cumulative level of volunteering and subsequent outcomes. Consequently, while there seems to be an association between the cumulative level of volunteering and the subsequent risk of hypertension, no such association was found for changes in volunteering. Similarly to Kim et al.,^6^ we also found that volunteering and/or charity work were not associated with subsequent depression, cognitive impairment or various physical health outcomes, including diabetes, stroke, cancer, heart attack, lung disease and chronic pain.

### Strengths and limitations

This study adds to the literature by leveraging a large sample and a longitudinal design, enabling the exploration of prospective associations, while considering various confounders, including prior engagement in volunteer and charity activities. This approach allowed us to examine reciprocal prospective associations in a single study, that is the effects of health and/or well-being on subsequent volunteer and/or charity activities and vice versa. We showed that volunteering and/or charity activities were more conducive to different outcomes than the other way around. However, the feedback loops between volunteer and/or charity activities and well-being outcomes as well as limitations in ADL and IADL were identified.

The main limitations of the study involve the fact that we could not directly disentangle charity work from voluntary work. Additionally, types of voluntary activities were not examined (e.g. money donation versus hard physical work), since only a general question about frequency of volunteering/charity work was available. Next, the reliance on self-reported data for health conditions and other aspects of well-being and daily life functioning might introduce social desirability bias and limit the accuracy of our results. However, the longitudinal design, controlling for pre-baseline outcomes and exposure, might mitigate these biases to some extent. Also, some reassurance is provided by prior studies that report a reasonable agreement between medical records and self-reported data on diseases. Finally, the data used were collected from middle-aged and older adults, which limited the generalizability of our results to this population.

## Conclusions

Voluntary and charity activities may contribute to the improvement of well-being and healthy aging among middle-aged and older Europeans. Nevertheless, comprehending the reciprocal relationship between volunteering and health and well-being can aid in developing strategies to encourage volunteering among this demographic, particularly those with specific health conditions. Additionally, these insights could inform the targeting of health and well-being promotion efforts toward volunteers.

## Supplementary Material

ckae014_Supplementary_Data

## Data Availability

This article uses data from SHARE Waves 4, 5, 7 and 8 (DOIs: 10.6103/SHARE.w4.710, 10.6103/SHARE.w5.710, 10.6103/SHARE.w7.710, 10.6103/SHARE.w8.100), see Börsch-Supan et al. (2013) for methodological details. These data are publicly available for a research purpose. The SHARE website (http://www.share-project.org/data-documentation.html) provides documentation of the study and access to the datasets. The SHARE data collection has been funded by the European Commission through FP5 (QLK6-CT-2001-00360), FP6 (SHARE-I3: RII-CT-2006-062193, COMPARE: CIT5-CT-2005-028857, SHARELIFE: CIT4-CT-2006-028812), FP7 (SHARE-PREP: GA N°211909, SHARE-LEAP: GA N°227822, SHARE M4: GA N°261982, DASISH: GA N°283646) and Horizon 2020 (SHARE-DEV3: GA N°676536, SHARE-COHESION: GA N°870628, SERISS: GA N°654221, SSHOC: GA N°823782) and by DG Employment, Social Affairs & Inclusion. Additional funding from the German Ministry of Education and Research, the Max Planck Society for the Advancement of Science, the U.S. National Institute on Aging (U01_AG09740-13S2, P01_AG005842, P01_AG08291, P30_AG12815, R21_AG025169, Y1-AG-4553-01, IAG_BSR06-11, OGHA_04-064, HHSN271201300071C) and from various national funding sources is gratefully acknowledged (see www.share-project.org). Börsch-Supan, A. (2020). Survey of Health, Ageing and Retirement in Europe (SHARE) Wave 4. Release version: 7.1.0. SHARE-ERIC. Data set. DOI: 10.6103/SHARE.w4.710 Börsch-Supan, A. (2020). Survey of Health, Ageing and Retirement in Europe (SHARE) Wave 7. Release version: 7.1.0. SHARE-ERIC. Data set. DOI: 10.6103/SHARE.w7.800 Börsch-Supan, A. (2021). Survey of Health, Ageing and Retirement in Europe (SHARE) Wave 8. Release version: 1.0.0. SHARE-ERIC. Data set. DOI: 10.6103/SHARE.w8.100 Key points Bi-directional longitudinal associations between volunteering and well-being and health are examined. Frequent volunteering is associated with subsequent greater emotional well-being and lower risks of activity limitations and Alzheimer’s disease. Health limitations and chronic diseases were found to hamper volunteering. Volunteering and well-being may have feedback effects. Policy actions could take the form of public awareness campaigns, partnerships with non-profits, development of volunteering opportunities and incentives or recognition programs.
