# Supplementary material for: Longitudinal reciprocal associations between volunteering, health and well-being: evidence for middle-aged and older adults in Europe
Source: Eur J Public Health. 2024 Feb 22;34(3):473–81. doi: 10.1093/eurpub/ckae014 (PMC11161165; doi:10.1093/eurpub/ckae014)
Supplement: ckae014_Supplementary_Data [file ckae014_supplementary_data.docx]

**Longitudinal associations between volunteering, health, and well-being. Evidence for middle-aged and older adults in Europe**

**Supplementary Material 1**

## **Measures**

**Emotional well-being outcomes.﻿** A sense of loneliness reflecting social isolation was measured using a 3-item loneliness scale, which is a short version of the UCLA Loneliness Scale (Hughes et al., 2004). A sample question from the scale is ’*How much of the time do you feel you lack companionship?’* The respondents provided information about the frequency of experiencing the feeling (1=hardly ever or never, 2=some of the time, and 3=often). The scale ranges from 3 to 9, with higher scores indicating higher levels of loneliness. Alternatively, loneliness was also measured using a single direct question about feeling lonely: ‘*How much of the time do you feel lonely?*’ (1=hardly ever or never, 2=some of the time, and 3=often).

The self-reported diagnosis of Alzheimer’s disease, dementia, or other serious memory impairment was also considered, as well as self-reported depression. To this end, the EURO-D geriatric depression scale (Guerra et al., 2015; Mehrbrodt et al., 2019; Prince et al., 1999) was used. This scale sums incidents of 12 depressive symptoms, including depression, pessimism, suicidality, guilt, disturbed sleep, limited or lost interest, irritability, limited or lost appetite, fatigue, reduced concentration (on reading or entertainment), limited or lost enjoyment, and tearfulness, and it ranges from 0 to 12. A score of 4 or higher is indicative of depression (Mehrbrodt et al., 2019). The internal consistency for this instrument was examined in numerous studies and Cronbach’s alpha ranged from 0.64 to 0.87 exceeding 0.70 in almost all of them (Guerra et al., 2015), while in SHARE, wave 8 it was 0.71.

Additionally, five single items related to well-being were considered separately from the CASP-12 questionnaire, which measures quality of life in early old age (Mehrbrodt et al., 2019), were considered separately. We focused on self-realization items (i.e., ‘*Future looks good*’ and ‘*I feel full of energy these days’)* as well as the assessment of eudaimonic and hedonic well-being, including both future time and past time assessment (i.e., ‘*On balance, I look back at my life with a sense of happiness*’, ‘*I look forward to each day’ and ‘I feel that my life has meaning*’). Respondents provided responses using a 4-point scale (often, sometimes, rarely, never).

**Daily life functioning outcomes.** Activities of daily living (ADL) and instrumental activities of daily living (IADL) were considered to assess daily functioning. The ADL consist of six daily self-care activities that are crucial for maintaining independence, such as dressing, walking, grooming, eating, transferring to bed, and toileting. The IADL consist of seven more complex activities such as preparing hot meals, shopping for groceries, making phone calls, taking medications, doing work in the house and garden, using a map to travel to an unknown location, and managing one’s money (e.g., paying bills, keeping track of expenses) (Chan et al., 2012; Mehrbrodt et al., 2019). In the analyses, two indicators of daily life functioning that measure the level of difficulty with ADL (Cronbach’s alpha=0.86; SHARE, wave 8) and IADL (Cronbach’s alpha=0.86; SHARE, wave 8), respectively, due to physical, mental, emotional, and memory problems, were used. The scores range from 0 to 6 or 7, respectively. Values above 0 indicate reported limitations with ADL and/or IADL.

**Physical health outcomes**

The following physical health outcomes were examined: heart attack, hypertension, high blood cholesterol, stroke, diabetes, chronic lung disease, and cancer. These variables accounted for both nonfatal and fatal medical conditions and were constructed using (i) self-reports of the presence or absence of a doctor’s diagnosis for these medical conditions from the main survey and (ii) exit interviews that report the cause of death.

Furthermore, we considered two additional variables: presence of impairing pain (‘*Do you feel troubled by pain?*’; yes or no), and whether respondents experienced at least one mobility, arm or fine motor limitation (yes or no).

**Cognitive impairment.** Cognitive impairment was considered using a measure of time orientation. Specifically, respondents were asked a series of questions about awareness of the current year, month, and day of the month, as well as about day of the week. This summary measure ranged from 0 to 4 (Dewey & Prince, 2015).

**Covariates.**

﻿Prior research suggested several main predictors of well-being and health, including demographic and family characteristics, work, education, and lifestyle (VanderWeele, 2017). Previous evidence also indicated that educational attainment, economic status, and health conditions contribute to the frequency of volunteering (Jensen et al., 2014). Additionally, personality traits have been shown to not only predict a predisposition to volunteer, but also moderate the association between volunteerism and health (Ackermann, 2019; King et al., 2015). Therefore, in this study, we adjusted for a number of variables (all self-reported and measured in the pre-baseline wave, that is, wave 4) that included demographic and socioeconomic factors, personality traits, health behaviors, and prior health history, as they could confound the examined associations. Demographic factors included gender (male and female), age (50–59, 60–69, 70–79, 80+), marital status (married and living with spouse, registered partnership, married but living separate from spouse, never married, divorced, widowed), highest educational attainment (according to the International Standard Classification of Education, version 1997 [ISCED-97], as available in the SHARE datasets), employment status (retired, employed or self-employed, unemployed, permanently sick of disabled, homemaker, other), and country. Socio-economic variables included annual personal income (after logarithmic transformation to account for skewness and outliers) and net financial assets of households (after logarithmic transformation to account for skewness and outliers). Personality traits included agreeableness, openness, neuroticism, conscientiousness, and extraversion, measured using the 10-item Big Five Inventory (BFI-10) (Rammstedt & John, 2007). The health behaviors accounted for: sports activity requiring a moderate level of effort (more than once a week, once a week, one to three times a month, hardly ever or never), alcohol consumption (almost every day, five or six days a week, three or four days a week, once or twice a week, once or twice a month, less than once a month, not at all in the last 6 months), and BMI.

﻿

**Supplementary Material 2**

**Table S1**

**Comparison of Outcomes Assessed at the Baseline Wave and the Outcome Wave. Survey of Health, Ageing and Retirement in Europe (SHARE), Middle-Aged and Older Adults Aged 50 and Over**

| Outcomes | Baseline Wave | | Outcome Wave | | *p*-value^a^ |
| --- | --- | --- | --- | --- | --- |
|  | % | Mean (SD) | % | Mean (SD) |  |
| Emotional well-being |  |  |  |  |  |
| Loneliness (3-item loneliness scale; 3-9) |  | 3.69 (1.20) |  | 3.85 (1.32) | <.001 |
| Alzheimer’s disease | 0.66 |  | 2.65 |  | <.001 |
| Depression (EURO-D≥4) | 23.39 |  | 26.36 |  | <.001 |
| Future looks good (1-4) |  | 3.15 (0.88) |  | 3.10 (0.89) | <.001 |
| I feel full of energy these days (1-4) |  | 3.25 (0.81) |  | 3.08 (0.87) | <.001 |
| On balance, I look back at my life with a sense of happiness (1-4) |  | 3.43 (0.74) |  | 3.44 (0.73) | .025 |
| I look forward to each day (1-4) |  | 3.54 (0.80) |  | 3.58 (0.73) | <.001 |
| I feel that my life has meaning (1-4) |  | 3.65 (0.66) |  | 3.60 (0.70) | <.001 |
| Daily life functioning |  |  |  |  |  |
| ADL  (at least 1 limitation) | 8.13 |  | 14.2 |  | <.001 |
| IADL  (at least 1 limitation) | 12.57 |  | 22.63 |  | <.001 |
| Physical health |  |  |  |  |  |
| Heart attack | 10.06 |  | 13.38 |  | <.001 |
| Hypertension | 39.64 |  | 45.36 |  | <.001 |
| High blood cholesterol | 23.04 |  | 25.11 |  | <.001 |
| Stroke | 2.73 |  | 4.22 |  | <.001 |
| Diabetes | 11.52 |  | 14.91 |  | <.001 |
| Cancer | 3.55 |  | 5.26 |  | <.001 |
| Chronic lung disease | 5.30 |  | 7.15 |  | <.001 |
| Pain | 42.54 |  | 46.68 |  | <.001 |
| Mobility (at least 1 limitation) | 45.65 |  | 54.18 |  | <.001 |
| Cognitive Impairment |  |  |  |  |  |
| Date orientation; 0-4 |  | 3.69 (0.81) |  | 3.53 (1.11) | .128 |

^a^Two-sided paired sample *t*-test

**Table S2**

Prospective Reciprocal Associations Between Emotional Well-Being, Physical Health, Daily Life Functioning, and Cognitive Impairment, and Subsequent Voluntary and/or Charity Work – Cross Lagged Panel Model. Survey of Health, Ageing and Retirement in Europe (SHARE), Adults aged 50 and Over (N=19,821)^a^

| Outcome | Voluntary and/or charity activity  (ref. = never) 🡪 subsequent PH-EWB-CI-DLF outcome | | | PH-EWB-CI-DLF indicator 🡪 subsequent voluntary and/or charity activity^c^ | |
| --- | --- | --- | --- | --- | --- |
|  | Statistic | Almost every month or less often | Almost every week or more often | Statistic | Ordinal logit model estimate |
| Emotional well-being |  |  |  |  |  |
| Loneliness (3-item loneliness scale) | $\beta^{b}$  (95% CI)  p-value | -0.021  (-0.069; 0.027)  0.397 | -0.021  (-0.058; 0.017)  0.280 | OR  (95% CI)  p-value | 0.922  (0.876; 0.971)  0.002 |
| Alzheimer’s disease | OR  (95% CI)  p-value | 0.649  (0.366; 1.150)  0.138 | 0.459 (0.312; 0.787)  0.003 | OR  (95% CI)  p-value | 1.091  (0.542; 2.200)  0.805 |
| Depression (EURO-D≥4) | RR  (95% CI)  p-value | 0.998  (0.864; 1.154)  0.981 | 1.032  (0.914; 1.166)  0.609 | OR  (95% CI)  p-value) | 0.831  (0.705; 0.981)  0.028 |
| Future looks good | $\beta^{b}$  (95% CI)  p-value | 0.047  (0.001; 0.094)  0.047 | 0.073  (0.023; 0.123)  0.005 | OR  (95% CI)  p-value | 1.094  (1.036; 1.154)  0.001 |
| I feel full of energy these days | $\beta^{b}$  (95% CI)  p-value | 0.039  (-0.024; 0.103)  0.226 | 0.121  0.083; 0.159)  <.001 | OR  (95% CI)  p-value | 1.166  (1.009; 1.227)  <.001 |
| On balance, I look back on my life with a sense of happiness | $\beta^{b}$  (95% CI)  p-value | 0.020  (-0.021; 0.061)  0.343 | 0.085  (0.048; 0.122)  <.001 | OR  (95% CI)  p-value | 0.012  (0.983; 1.080)  0.208 |
| I look forward to each day | $\beta^{b}$  (95% CI)  p-value | 0.017  (-0.026; 0.060)  0.427 | 0.042  (0.002; 0.083)  0.039 | OR  (95% CI)  p-value | 0.991  (0.952; 1.031)  0.645 |
| I feel that my life has meaning | $\beta^{b}$  (95% CI)  p-value | 0.043  (0.005; 0.081)  0.027 | 0.059  (0.000; 0.117)  0.048 | OR  (95% CI)  p-value | 1.088  (1.036; 1.142)  0.001 |
| Daily life functioning |  |  |  |  |  |
| ADL  (at least 1 limitation) | OR  (95% CI)  p-value | 0.774  (0.630; 0.950)  0.014 | 0.662  (0.542; 0. 810)  <.001 | OR  (95% CI)  p-value | 0.720  (0.559; 0.929)  0.012 |
| IADL  (at least 1 limitation) | RR  (95% CI)  p-value | 0.766  (0.651; 0.902)  0.001 | 0.750  (0.636; 0.884)  0.001 | OR  (95% CI)  p-value | 0.791  (0.648; 0.967)  0.022 |
| Physical health |  |  |  |  |  |
| Heart attack | RR  (95% CI)  p-value | 0.850  (0.648; 1.115)  0.240 | 0.931  (0.780; 1.110)  0.424 | OR  (95% CI)  p-value | 1.003  (0.855; 1.178)  0.967 |
| Hypertension | RR  (95% CI)  p-value | 1.028  (0.920; 1.150)  0.622 | 1.022  (0.866; 1.206)  0.798 | OR  (95% CI)  p-value | 0.894  (0.804; 0.994)  0.038 |
| High blood cholesterol | RR  (95% CI)  p-value | 1.084  (0.986; 1.192)  0.096 | 0.990  (0.848; 1.156)  0.901 | OR  (95% CI)  p-value | 0.825  (0.716; 0.949)  0.007 |
| Stroke | OR  (95% CI)  p-value | 0.657  (0.415; 1.040)  0.073 | 0.935  (0.716; 1.220)  0.619 | OR  (95% CI)  p-value | 0.809  (0.620; 1.055)  0.117 |
| Diabetes | RR  (95% CI)  p-value | 0.978  (0.786; 1.217)  0.841 | 0.857  (0.723; 1.016)  0.075 | OR(95% CI)  p-value | 0.996  (0.797; 1.246)  0.975 |
| Cancer | OR  (95% CI)  p-value | 0.997  (0.790; 1.260)  0.981 | 0.904  (0.729; 1.120)  0.354 | OR(95% CI)  p-value | 01109  (0.877; 1.405)  0.387 |
| Chronic lung disease | OR  (95% CI)  p-value | 0.970  (0.729; 1.291)  0.835 | 1.188  (0.950; 1.485)  0.131 | OR(95% CI)  p-value | 0.637  (0.474; 0.857)  0.003 |
| Pain | RR  (95% CI)  p-value | 0.952  (0.822; 1.103)  0.515 | 0.948  (0.852; 1.054)  0.322 | OR(95% CI)  p-value | 0.902  (0.817; 0.995)  0.039 |
| Mobility (at least 1 limitation) | RR  (95% CI)  p-value | 1.001  (0.858; 1.168)  0.989 | 0.966  (0.854; 1.092)  0.577 | OR(95% CI)  p-value | 0.895  (0.802; 0.998)  0.047 |
| Cognitive Impairment |  |  |  |  |  |
| Date Orientation | $\beta^{b}$  (95% CI)  p-value | 0.021  (-0.016; 0.059)  0.267 | 0.052  (0.002; 0.102)  0.043 | OR(95% CI)  p-value | 1.026  (0.961; 1.099)  0.445 |

*Note:* CI, confidence interval; OR, odds ratio; RR, risk ratio; ADL, activities of daily living; IADL, instrumental activities of daily living.

^a^ Missing covariate variables were imputed using chained equations (10 sets of imputed data were generated). All the models were controlled for participant demographics: age, gender, marital status, educational attainment, labor market status, country; socio-economic factors: annual personal income, household net financial assets; health behaviors such as BMI, alcohol consumption, and sports activity; and personality traits: agreeableness, openness, conscientiousness, neuroticism, extraversion. Each model was also adjusted for prior values of the health, daily life functioning and cognitive impairment outcomes and for prior values of the exposure variable. In the model for pain, the prior values of this outcome are not controlled for due to lack of this outcome measured in wave 4. Cross-lagged panel model was estimated. The p-value cut-off for Bonferroni correction = 0.05/21 indicators =0.0024); significant estimates at traditional p<0.05 are bolded.

^b^ All the continuous outcomes were standardized (mean = 0, standard deviation, 1), and β was the standardized effect size.

^c^ Voluntary and/or charity activity when modelled as an outcome entered the analysis as a continuous variable.

**Table S3**

Prospective Reciprocal Associations Between Emotional Well-Being, Physical Health, Daily Life Functioning, and Cognitive Impairment, and Subsequent Voluntary and/or Charity Work – Cross Lagged Panel Model with Continuous Voluntary and/or Charity Work Variable. Survey of Health, Ageing and Retirement in Europe (SHARE), Adults aged 50 and Over (N=19,821)^a^

| Outcome | Voluntary and/or charity activity  🡪 subsequent PH-EWB-CI-DLF outcome | | PH-EWB-CI-DLF indicator 🡪 subsequent voluntary and/or charity activity^c^ | |
| --- | --- | --- | --- | --- |
| Emotional well-being |  |  |  |  |
| Loneliness (3-item loneliness scale) | $\beta^{b}$  (95% CI)  p-value | -0.010  (-0.027; 0.006)  0.207 | $\beta^{b}$  (95% CI)  p-value | -0.015  (-0.027; -0.003)  0.017 |
| Alzheimer’s disease | OR  (95% CI)  p-value | 0.820  (0.708; 0.949)  0.008 | $\beta^{b}$  (95% CI)  p-value | 0.070  (-0.090; 0.230)  0.383 |
| Depression (EURO-D≥4) | RR  (95% CI)  p-value | 1.006  (0.969; 1.004)  0.763 | $\beta^{b}$  (95% CI)  p-value) | -0.044  (-0.080; -0.008)  0.017 |
| Future looks good | $\beta^{b}$  (95% CI)  p-value | 0.027  (0.012; 0.042)  <.001 | $\beta^{b}$  (95% CI)  p-value | 0.029  (0.009; 0.048)  0.004 |
| I feel full of energy these days | $\beta^{b}$  (95% CI)  p-value | 0.037  (0.025; 0.049)  <.001 | $\beta^{b}$  (95% CI)  p-value | 0.037  (0.023; 0.052)  <.001 |
| On balance, I look back on my life with a sense of happiness | $\beta^{b}$  (95% CI)  p-value | 0.027  (0.015; 0.039)  <.001 | $\beta^{b}$  (95% CI)  p-value | 0.014  (0.001; 0.027)  0.030 |
| I look forward to each day | $\beta^{b}$  (95% CI)  p-value | 0.018  (0.005; 0.031)  0.008 | $\beta^{b}$  (95% CI)  p-value | -0.001  (-0.012; 0.011)  0.884 |
| I feel that my life has meaning | $\beta^{b}$  (95% CI)  p-value | 0.023  (0.004; 0.042)  0.019 | $\beta^{b}$  (95% CI)  p-value | 0.015  (0.003; 0.028)  0.013 |
| Daily life functioning |  |  |  |  |
| ADL  (at least 1 limitation) | OR  (95% CI)  p-value | 0.885  (0.6825; 0.949)  0.001 | $\beta^{b}$  (95% CI)  p-value | -0.048  (-0.114; 0.018)  0.151 |
| IADL  (at least 1 limitation) | RR  (95% CI)  p-value | 0.906  (0.863; 0.951)  <.001 | ($\beta^{b}$  (95% CI)  p-value | -0.034  (-0.081; -0.014)  0.167 |
| Physical health |  |  |  |  |
| Heart attack | RR  (95% CI)  p-value | 0.964  (0.900; 1.032)  0.292 | $\beta^{b}$  (95% CI)  p-value | 0.011  (-0.030; 0.052)  0.604 |
| Hypertension | RR  (95% CI)  p-value | 1.006  (0.954; 1.060)  0.829 | $\beta^{b}$  (95% CI)  p-value | -0.055  (-0.092; -0.019)  0.003 |
| High blood cholesterol | RR  (95% CI)  p-value | 1.001  (0.954; 1.051)  0.954 | $\beta^{b}$  (95% CI)  p-value | -0.027  (-0.058; -0.004)  0.087 |
| Stroke | OR  (95% CI)  p-value | 0.940  (0.846; 1.045)  0.251 | $\beta^{b}$  (95% CI)  p-value | -0.047  (-0.128; 0.034)  0.250 |
| Diabetes | RR  (95% CI)  p-value | 0.966  (0.917; 1.018)  0.197 | $\beta^{b}$  (95% CI)  p-value | 0.002  (-0.045; 0.050)  0.927 |
| Cancer | OR  (95% CI)  p-value | 0.973  (0.894; 1.059)  0.526 | $\beta^{b}$  (95% CI)  p-value | 0.012  (-0.071; 0.095)  0.772 |
| Chronic lung disease | OR  (95% CI)  p-value | 1.052  (0.978; 1.132)  0.174 | $\beta^{b}$  (95% CI)  p-value | -0.100  (-0.163; -0.038)  0.002 |
| Pain | RR  (95% CI)  p-value | 0.952  (0.822; 1.103)  0.515 | $\beta^{b}$  (95% CI)  p-value | -0.032  (-0.065; 0.002)  0.061 |
| Mobility (at least 1 limitation) | RR  (95% CI)  p-value | 0.991  (0.947; 1.037)  0.692 | $\beta^{b}$  (95% CI)  p-value | -0.033  (-0.062; -0.004)  0.025 |
| Cognitive Impairment |  |  |  |  |
| Date Orientation | $\beta^{b}$  (95% CI)  p-value | 0.016  (-0.00; 0.032)  0.052 | $\beta^{b}$  (95% CI)  p-value | 0.004  (-0.011; 0.018)  0.616 |

*Note:* CI, confidence interval; OR, odds ratio; RR, risk ratio; ADL, activities of daily living; IADL, instrumental activities of daily living.

^a^ Missing covariate variables were imputed using chained equations (10 sets of imputed data were generated). All the models were controlled for participant demographics: age, gender, marital status, educational attainment, labor market status, country; socio-economic factors: annual personal income, household net financial assets; health behaviors such as BMI, alcohol consumption, and sports activity; and personality traits: agreeableness, openness, conscientiousness, neuroticism, extraversion. Each model was also adjusted for prior values of the health, daily life functioning and cognitive impairment outcomes and for prior values of the exposure variable. In the model for pain, the prior values of this outcome are not controlled for due to lack of this outcome measured in wave 4. Cross-lagged panel model was estimated. The p-value cut-off for Bonferroni correction = 0.05/21 indicators =0.0024); significant estimates at traditional p<0.05 are bolded.

^b^ All the continuous indicators were standardized (mean = 0, standard deviation, 1), and β was the standardized effect size.

**Table S4**

**Prospective Associations Between Voluntary and/or Charity Work and Emotional Well-Being, Physical Health, Daily Life Functioning, and Cognitive Impairment. Survey of Health, Ageing and Retirement in Europe (SHARE), Adults aged 50 and Over; Complete Case Scenario^a^.**

| Outcome | Voluntary and/or charity activity  (ref. = never) 🡪 subsequent PH-EWB-CI-DLF outcome | | | PH-EWB-CI-DLF indicator 🡪 subsequent voluntary and/or charity activity^c^ | |
| --- | --- | --- | --- | --- | --- |
|  | Statistic^b,c^ | Almost every month or less often | Almost every week or more often | Statistic | Ordinal logit model estimate |
| Emotional well-being |  |  |  |  |  |
| Loneliness (3-item loneliness scale) | $\beta^{b}$  (95% CI)  p-value  N=8,204 | -0.029  (-0.112; 0.055)  0.465 | -0.001  (-0.081; 0.079)  0.972 | OR  (95% CI)  p-value  N=13,599 | 0.935  (0.878; 0.996)  0.037 |
| Alzheimer’s disease | OR  (95% CI)  p-value  N=8,160 | 0.840  (0.295; 2.390)  0.744 | 0.848  (0.411; 1.747)  0.655 | $\mathrm{OR}$  (95% CI)  p-value  N=13,666 | 1.126  (0.557; 2.268)  0.741 |
| Depression (EURO-D≥4) | RR  (95% CI)  p-value  N=8,134 | 1.031  (0.920; 1.156)  0.599 | 1.041  (0.887; 1.221)  0.623 | OR  (95% CI)  p-value)  N=13,547 | 0.879  (0.727; 1.063)  0.183 |
| Future looks good | $\beta^{b}$  (95% CI)  p-value  N=8,147 | 0.026  (-0.055; 0.108)  0.490 | 0.051  (0.005; 0.098)  0.034 | OR  (95% CI)  p-value  N=13,516 | 1.094  (1.030; 1.161)  0.004 |
| I feel full of energy these days | $\beta^{b}$  (95% CI)  p-value  N=8,221 | 0.046  (-0.038; 0.130)  0.253 | 0.141  (0.071; 0.210)  0.001 | OR  (95% CI)  p-value  N=13,620 | 1.141  (1.076; 1.209)  <.001 |
| On balance, I look back on my life with a sense of happiness | $\beta^{b}$  (95% CI)  p-value  N=8,201 | 0.013  (-0.054; 0.080)  0.680 | 0.076  (0.028; 0.125)  0.005 | OR  (95% CI)  p-value  N=13,580 | 0.989  (0.944; 1.036)  0.640 |
| I look forward to each day | $\beta^{b}$  (95% CI)  p-value  N=8,193 | -0.013  (-0.072; 0.046)  0.630 | 0.046  (-0.031; 0.124)  0.216 | OR  (95% CI)  p-value  N=13,578 | 0.975  (0.935; 1.018)  0.252 |
| I feel that my life has meaning | $\beta^{b}$  (95% CI)  p-value  N= 8,159 | 0.034  (-0.009; 0.078)  0.113 | 0.023  (-0.030; 0.076)  0.360 | OR  (95% CI)  p-value  N=13,552 | 1.014  (1.026; 1.119)  0.002 |
| Daily life functioning |  |  |  |  |  |
| ADL  (at least 1 limitation) | OR  (95% CI)  p-value  N=8,331 | 0.935  (0.730; 1.196)  0.590 | 0.569  (0.421;0.771)  <.001 | OR  (95% CI)  p-value  N=13,672 | 0.698  (0.534; 0.913)  0.009 |
| IADL  (at least 1 limitation) | RR  (95% CI)  p-value  N= 8,331 | 0.834  (-0.048; -0.001)  0.077 | 0.825  (0.670; 1.014)  0.068 | OR  (95% CI)  p-value  N=13,672 | 0.762  (0.607; 0.956)  0.019 |
| Physical health |  |  |  |  |  |
| Heart attack | RR  (95% CI)  p-value  N=8,338 | 0.854  (0.657; 1.109)  0.236 | 0.992  (0.788; 1.249)  0.948 | OR  (95% CI)  p-value  N=13,666 | 1.030  (0.885; 1.200)  0.702 |
| Hypertension | RR  (95% CI)  p-value  N=8,338 | 1.003  (0.939; 1.071)  0.935 | 0.962  (0.892; 1.038)  0.318 | OR  (95% CI)  p-value  N=13,666 | 0.871  (0.772; 0.982)  0.024 |
| High blood cholesterol | RR  (95% CI)  p-value  N=8,338 | 1.128  (1.034; 1.232)  0.007 | 0.989  (0.865; 1.130)  0.870 | OR  (95% CI)  p-value  N=13,666 | 0.857  (0.715; 1.012)  0.069 |
| Stroke | OR  (95% CI)  p-value  N=8,287 | 0.729  (0.382; 1.139)  0.337 | 1.130  (0.972; 1.314)  0.112 | OR  (95% CI)  p-value | 0.986  (0.745; 1.305)  0.921 |
| Diabetes | RR  (95% CI)  p-value  N=8,338 | 0.967  (0.847; 1.104)  0.617 | 0.617  (0.745; 1.110)  0.350 | OR  (95% CI)  p-value  N=13,666 | 0.948  (0.719; 1.250)  0.705 |
| Cancer | OR  (95% CI)  p-value  N=8,330 | 1.015  (0.803; 1.283)  0.904 | 0.945  (0.630; 1.418)  0.786 | OR  (95% CI)  p-value  n=13,666 | 1.149  (0.910; 1.449)  0.243 |
| Chronic lung disease | OR  (95% CI)  p-value  N=19,821 | 1.032  (0.644; 1.654)  0.896 | 1.353  (0.903; )  0.143 | OR  (95% CI)  p-value | 0.610  (0.435; 0.856)  0.004 |
| Pain | RR  (95% CI)  p-value  N=8,328 | 0.970  (0.898; 1.048)  0.442 | 1.000  (0.918; 1.089)  0.996 | OR  (95% CI)  p-value  N=13,672 | 0.905  (0.794; 1.031)  0.133 |
| Mobility | RR  (95% CI)  p-value  N=8,332 | 1.043  (0.966; 1.127)  0.283 | 1.010  (0.971; 1.049)  0.631 | OR  (95% CI)  p-value  N=13,672 | 0.919  (0.802; 1.054)  0.229 |
| Cognitive Impairment |  |  |  |  |  |
| Date Orientation | $\beta^{b}$  (95% CI)  p-value  N=8,336 | 0.013  (-0.037; 0.063)  0.587 | 0.008  (-0.067; 0.083)  0.823 | OR  (95% CI)  p-value  N=8,272) | 1.001  (0.907; 1.105)  0.989 |

CI, confidence interval; OR, odds ratio; RR, risk ratio; ADL, activities of daily living; IADL, instrumental activities of daily living

^a^ All the models were controlled for participant demographics: age, gender, marital status, educational attainment, labor market status, country; socio-economic factors: annual personal income, household net financial assets; health behaviors such as BMI, alcohol consumption, and sports activity; and personality traits: agreeableness, openness, conscientiousness, neuroticism, extraversion. Each model was also adjusted for prior values of the 21 outcome variables, all simultaneously in each regression model and for prior value of the exposure variable.

^b^ All the continuous outcomes were standardized (mean = 0, standard deviation, 1), and β was the standardized effect size.

^c^ The p-value cut-off for Bonferroni correction = 0.05/21 indicators =0.0024).

**Table S5**

**Prospective Associations Between Voluntary and/or Charity Work and Emotional Well-Being, Physical Health, Daily Life Functioning, and Cognitive Impairment. Survey of Health, Ageing and Retirement in Europe (SHARE), Adults aged 50 and Over; Limited Set of Control Variables (N=19,821)^a^.**

| Outcome | Statistics^b,c^ | Voluntary and/or charity activity  (ref. = never) 🡪 subsequent PH-EWB-CI-DLF outcome | | | Statistics^b,c^ | PH-EWB-CI-DLF indicator 🡪 subsequent voluntary and/or charity activity^c^ | |
| --- | --- | --- | --- | --- | --- | --- | --- |
|  |  | Model | Almost every month or less often | Almost every week or more often |  | Model | Ordinal logit model estimate |
| Emotional well-being |  |  |  |  |  |  |  |
| Loneliness (3-item loneliness scale) | $\beta^{b}$  (95% CI)  p-value | Model 0 | -0.023  (-0.070; 0.025)  0.316 | -0.019  (-0.061; 0.023)  0.341 | OR  (95% CI)  p-value | Model 0 | 0.939  (0.892; 0.989)  0.017 |
|  |  | Model 1 | -0.085  (-0.149; -0.020)  0.014 | -0.078  (-0.126; -0.030)  0.005 |  | Model 1 | 0.819  (0.766; 0.877)  <.001 |
|  |  | Model 2 | -0.050  (-0.106; 0.005)  0.072 | -0.045  (-0.087; -0.004)  0.034 |  | Model 2 | 0.882  (0.833; 0.934)  <.001 |
|  |  | Model 3 | -0.026  (-0.073; 0.022)  0.256 | -0.026  (-0.064; 0.012)  0.157 |  | Model 3 | 0.913  (0.867; 0.961)  0.001 |
| Alzheimer’s disease | OR  (95% CI)  p-value | Model 0 | 0.639  (0.364; 1.121)  0.118 | 0.543  (0.351; 0.838)  0.006 | OR  (95% CI)  p-value | Model 0 | 1.099  (0.536; 1.253)  0.796 |
|  |  | Model 1 | 0.560  (0.316;0.990)  0.046 | 0.465  (0.298; 0.726)  0.001 |  | Model 1 | 0.682  (0.373; 1.247)  0.212 |
|  |  | Model 2 | 0.591  (0.337; 1.036)  0.066 | 0.488  (0.309; 0.769)  0.002 |  | Model 2 | 0.809  (0.415; 1.577)  0.532 |
|  |  | Model 3 | 0.629  (0.356; 1.111)  0.110 | 0.510  (0.323; 0.806)  0.004 |  | Model 3 | 1.051  (0.530; 2.085)  0.885 |
| Depression (EURO-D≥4) | RR  (95% CI)  p-value | Model 0 | 0.987  (0.907; 1.074)  0.762 | 1.033  (0.953; 1.119)  0.435 | OR  (95% CI)  p-value | Model 0 | 0.860  (0.727; 1.018)  0.079 |
|  |  | Model 1 | 0.866  (0.771; 0.972)  0.015 | 0.904  (0.805; 1.014)  0.086 |  | Model 1 | 0.693  (0.611; 0.787)  <.001 |
|  |  | Model 2 | 0.919  (0.825; 1.023)  0.123 | 0.952  (0.858; 1.055)  0.347 |  | Model 2 | 0.761  (0.659; 0.878)  <.001 |
|  |  | Model 3 | 0.973  (0.887;1.068)  0.568 | 1.001  (0.926; 1.081)  0.779 |  | Model 3 | 0.811  (0.689; 0.954)  0.012 |
| Future looks good | $\beta^{b}$  (95% CI)  p-value | Model 0 | 0.052  (0.005; 0.100)  0.034 | 0.070  (0.020; 0.121)  0.010 | OR  (95% CI)  p-value | Model 0 | 1.083  (1.024; 1.145)  0.005 |
|  |  | Model 1 | 0.142  (0.073; 0.210)  0.001 | 0.166  (0.956; 0.235)  <.001 |  | Model 1 | 1.284  (1.214; 1.358)  <.001 |
|  |  | Model 2 | 0.096  (0.041; 0.150)  0.003 | 0.124  (0.069; 0.179)  <.001 |  | Model 2 | 0.072  (1.125; 1.257)  <.001 |
|  |  | Model 3 | 0.057  (0.009; 0.105)  0.024 | 0.082  (0.032; 0.132)  0.004 |  | Model 3 | 1.104  (1.045; 1.165)  <.001 |
| I feel full of energy these days | $\beta^{b}$  (95% CI)  p-value | Model 0 | 0.046  (-0.034; 0.126)  0.226 | 0.114  (0.065; 0.164)  <.001 | OR  (95% CI)  p-value | Model 0 | 1.149  (1.093; 1.210)  <.001 |
|  |  | Model 1 | 0.138  (0.046; 0.229)  0.007 | 0.227  (0.154; 0.299)  <.001 |  | Model 1 | 1.310  (1.218; 1.409)  <.001 |
|  |  | Model 2 | 0.084  (-0.002;0.169)  0.054 | 0.179  (0.126; 0.233)  <.001 |  | Model 2 | 1.234  (1.151; 1.323)  <.001 |
|  |  | Model 3 | 0.053  (-0.023; 0.130)  0.150 | 0.128  (0.781; 0.178)  <.001 |  | Model 3 | 1.176  (1.114; 1.242)  <.001 |
| On balance, I look back on my life with a sense of happiness | $\beta^{b}$  (95% CI)  p-value | Model 0 | 0.022  (-0.028; 0.072)  0.349 | 0.074  (0.031; 0.117)  0.003 | OR  (95% CI)  p-value | Model 0 | 1.007  (0.962; 1.055)  0.758 |
|  |  | Model 1 | 0.119  (0.045; 0.192)  0.004 | 0.186  (0.121; 0.251)  <.001 |  | Model 1 | 1.170  (1.108; 1.235)  <.001 |
|  |  | Model 2 | 0.073  (0.012; 0.134)  0.022 | 0.141  (0.090; 0.192)  <.001 |  | Model 2 | 1.103  (1.046; 1.163)  <.001 |
|  |  | Model 3 | 0.027  (-0.025; 0.078)  0.275 | 0.084  (0.041; 0.127)  0.001 |  | Model 3 | 1.037  (0.987; 1.088)  0.147 |
| I look forward to each day | $\beta^{b}$  (95% CI)  p-value | Model 0 | 0.022  (-0.028; 0.071)  0.344 | 0.039  (-0.010; 0.088)  0.109 | OR  (95% CI)  p-value | Model 0 | 0.977  (0.942; 1.016)  0.217 |
|  |  | Model 1 | 0.114  (0.042; 0.185)  0.005 | 0.119  (-0.013; 0.251)  0.073 |  | Model 1 | 1.056  (0.986; 1.131)  0.119 |
|  |  | Model 2 | 0.069  (0.005; 0.133)  0.038 | 0.075  (-0.031; 0.181)  0.148 |  | Model 2 | 1.015  (0.963; 1.069)  0.587 |
|  |  | Model 3 | 0.031  (-.021; 0.083)  0.202 | 0.053  (0.003; 0.104)  0.040 |  | Model 3 | 0.996  (0.958; 1.036)  0.860 |
| I feel that my life has meaning | $\beta^{b}$  (95% CI)  p-value | Model 0 | 0.040  (-0.012; 0.092)  0.117 | 0.053  (-0.007; 0.112)  0.077 | OR  (95% CI)  p-value | Model 0 | 1.078  (1.026; 1.133)  0.003 |
|  |  | Model 1 | 0.129  (0.068; 0.190)  0.001 | 0.149  (0.059; 0.239)  0.004 |  | Model 1 | 1.221  (1.141; 1. 306)  <.001 |
|  |  | Model 2 | 0.089  (0.345; 0.142)  0.005 | 0.109  (0.032; 0.186)  0.010 |  | Model 2 | 1.153  (.089; 1.221)  <.001 |
|  |  | Model 3 | 0.048  (-0.004; 0.996)  0.065 | 0.066  (0.004; 0.129)  0.039 |  | Model 3 | 1.094  (1.041; 1.149)  <.001 |
| Daily life functioning |  |  |  |  |  |  |  |
| ADL  (at least 1 limitation) | OR  (95% CI)  p-value | Model 0 | 0.791  (0.645; 0.970)  0.024 | 0.683  (0.575; 0. 812)  <.001 | OR  (95% CI)  p-value | Model 0 | 0.737  (0.569; 0.953)  0.020 |
|  |  | Model 1 | 0.692  (0.573; 0.835)  <.001 | 0.585  (0.498; 0.686)  <.001 |  | Model 1 | 0.565  (0.437; 0.729)  <.001 |
|  |  | Model 2 | 0.754  (0.624; 0.911)  0.004 | 0.626  (0.534; 0.735)  <.001 |  | Model 2 | 0.657  (0.507; 0. 850)  0.001 |
|  |  | Model 3 | 0.772  (0.633; 0.941)  0.011 | 0.646  (0.545; 0.767)  <.001 |  | Model 3 | 0.689  (0.534; 0.889)  0.004 |
| IADL  (at least 1 limitation) | RR  (95% CI)  p-value | Model 0 | 0.833  (0.745; 0.930)  0.001 | 0.826  (0.749; 0.911)  <.001 | OR  (95% CI)  p-value | Model 0 | 0.811  (0.661; 0.993)  0. 043 |
|  |  | Model 1 | 0.741  (0.649; 0.847)  <.001 | 0.739  (0.660; 0.826)  <.001 |  | Model 1 | 0.635  (0.537; 0.752)  <.001 |
|  |  | Model 2 | 0.792  (0.704; 0.890)  <.001 | 0.777  (0.695; 0.868)  <.001 |  | Model 2 | 0.722  (0.608; 0.857)  <.001 |
|  |  | Model 3 | 0.757  (0.649; 0.884)  <.001 | 0.744  (0.639; 0.867)  <.001 |  | Model 3 | 0.763  (0.629; 0.927)  0.006 |
| Physical health |  |  |  |  |  |  |  |
| Heart attack | RR  (95% CI)  p-value | Model 0 | 0.865  (0.689; 1.087)  0.214 | 0.950  (0.837; 1.079)  0.432 | OR  (95% CI)  p-value | Model 0 | 1.010  (0.860; 1.182)  0.905 |
|  |  | Model 1 | 0.814  (0.647; 1.026)  0.080 | 0.873  (0.772; 0.987)  0.030 |  | Model 1 | 0.854  (0.718; 1.016)  0.074 |
|  |  | Model 2 | 0.849  (0.673; 1.066)  0.156 | 0.879  (0.808; 1.005)  0.061 |  | Model 2 | 0.942  (0.796; 1.115)  0.490 |
|  |  | Model 3 | 0.858  (0.685; 1.074)  0.181 | 0.928  (0.816; 1.055)  0.254 |  | Model 3 | 0.985  (0.841; 1.155)  0.856 |
| Hypertension | RR  (95% CI)  p-value | Model 0 | 1.005  (0.965; 1.046)  0.813 | 1.010  (0.936; 1.089)  0.806 | OR  (95% CI)  p-value | Model 0 | 0.897  (0.807; 0.996)  0.042 |
|  |  | Model 1 | 0.958  (0.911; 1.007)  0.089 | 0.943  (0.861; 1.032)  0.179 |  | Model 1 | 0.772  (0.687; 0.868)  <.001 |
|  |  | Model 2 | 0.985  (0.942; 1.031)  0.516 | 0.970  (0.886; 1.061)  0.499 |  | Model 2 | 0.827  (0.733; 0.932)  0.002 |
|  |  | Model 3 | 1.006  (0.966; 1.048)  0.765 | 1.010  (0.937; 1.088)  0.796 |  | Model 3 | 0.891  (0.801; 0.990)  0.032 |
| High blood cholesterol | RR  (95% CI)  p-value | Model 0 | 1.064  (1.004; 1.128)  0.037 | 1.006  (0.899; 1.126)  0.918 | OR  (95% CI)  p-value | Model 0 | 0.829  (0.721; 0.953)  0.008 |
|  |  | Model 1 | 1.028  (0.963; 1.099)  0.407 | 0.959  (0.842; 1.091)  0.521 |  | Model 1 | 0.764  (0.663; 0.880)  <.001 |
|  |  | Model 2 | 1.036  (0.974; 1.101)  0.260 | 0.959  (0.784; 1.121)  0.497 |  | Model 2 | 0.789  (0.686; 0.910)  0.001 |
|  |  | Model 3 | 1.068  (1.006; 1.134)  0.032 | 1.007  (0.902; 1.124)  0.899 |  | Model 3 | 0.818  (0.713; 0.940)  0.005 |
| Stroke | OR  (95% CI)  p-value | Model 0 | 0.644  (0.413; 1.004)  0.053 | 0.910  (0.690; 1.199)  0.499 | OR  (95% CI)  p-value | Model 0 | 0.812  (0.620; 1.063)  0.129 |
|  |  | Model 1 | 0.575  (0.377; 0.877)  0.010 | 0.800  (0.607; 1.054)  0.112 |  | Model 1 | 0.728  (0.564; 0.940)  0.015 |
|  |  | Model 2 | 0.621  (0.403; 0.957)  0.031 | 0.856  (0.643; 1.139)  0.284 |  | Model 2 | 0.784  (0.601; 1.024)  0.074 |
|  |  | Model 3 | 0.635  (0.413; 0.977)  0.039 | 0.883  (0.667; 1.169)  0.382 |  | Model 3 | 0.789  (0.607; 1.026)  0.076 |
| Diabetes | RR  (95% CI)  p-value | Model 0 | 0.978  (0.864; 1.107)  0.729 | 0.916  (0.829; 1.011)  0.080 | OR  (95% CI)  p-value | Model 0 | 0.995  (0.798; 1.241)  0.966 |
|  |  | Model 1 | 0.903  (0.801; 1.018)  0.096 | 0.829  (0.760; 0.905)  <.001 |  | Model 1 | 0.803  (0.659; 0.979)  0.030 |
|  |  | Model 2 | 0.960  (0.857; 1.075)  0.473 | 0.861  (0.790; 0.939)  0.001 |  | Model 2 | 0. 921  (0.745; 1.140)  0.451 |
|  |  | Model 3 | 0.981  (0868; 1.109)  0.760 | 0.910  (0.825; 1.003)  0.058 |  | Model 3 | 0.986  (0.792; 1.228)  0.901 |
| Cancer | OR  (95% CI)  p-value | Model 0 | 1.017  (0.801; 1.292)  0.888 | 0.934  (0.748; 1.165)  0.543 | OR  (95% CI)  p-value | Model 0 | 1.114  (0.882; 1.406)  0.364 |
|  |  | Model 1 | 1.013  (0.800; 1.284)  0.913 | 0.962  (0.779; 1.187)  0.715 |  | Model 1 | 1.042  (0.835; 1.301)  0.713 |
|  |  | Model 2 | 1.028  (0.809; 1.307)  0.819 | 0.967  (0.785; 1.190)  0.751 |  | Model 2 | 1.076  (0.857; 1.350)  0.527 |
|  |  | Model 3 | 1.010  (0.799; 1.276)  0.935 | 0.926  (0.751; 1.142)  0.470 |  | Model 3 | 1.097  (0.867; 1.387)  0.443 |
| Chronic lung disease | OR  (95% CI)  p-value | Model 0 | 0.965  (0.724; 1.286)  0.807 | 1.234  (0.990; 1.539)  0.062 | OR  (95% CI)  p-value | Model 0 | 0.640  (0.477; 0.858)  0.003 |
|  |  | Model 1 | 0.940  (0.716; 1.234)  0.657 | 1.201  (0.975;1.480)  0.085 |  | Model 1 | 0.612  (0.462; 0.812)  0.001 |
|  |  | Model 2 | 0.967  (0.740; 1.263)  0.806 | 1.204  (0.980; 1.482)  0.077 |  | Model 2 | 0.637  (0.477; 0.852)  0.002 |
|  |  | Model 3 | 0.941  (0.710; 1.245)  0.672 | 1.185  (0.954; 1.472)  0.125 |  | Model 3 | 0.625  (0.467; 0.836)  0.002 |
| Pain | RR  (95% CI)  p-value | Model 0 | 0.963  (0.900; 1.030)  0.273 | 0.983  (0.926; 1.044)  0.581 | OR  (95% CI)  p-value | Model 0 | 0.906  (0.821; 1.001)  0.051 |
|  |  | Model 1 | 0.954  (0.924; 0.984)  0.003 | 0.963  (0.932; 0.995)  0.025 |  | Model 1 | 0.696  (0.627; 0.773)  <.001 |
|  |  | Model 2 | 0.928  (0.865; 0.996)  0.038 | 0.942  (0.874; 1.015)  0.115 |  | Model 2 | 0.803  (0.723; 0. 890)  <.001 |
|  |  | Model 3 | 0.982  (0.958; 1.007)  0.163 | 0.991  (0.968; 1.016)  0.490 |  | Model 3 | 0.863  (0.790; 0.942)  0.001 |
| Mobility | RR  (95% CI)  p-value | Model 0 | 0.997  (0.942; 1.056)  0.930 | 0.997  (0.954; 1.042)  0.893 | OR  (95% CI)  p-value | Model 0 | 0.904  (0.809; 1.011)  0.077 |
|  |  | Model 1 | 0.978  (0.948; 1.008)  0.145 | 0.974  (0.951; 0.997)  0.030 |  | Model 1 | 0.738  (0.669; 0.816)  <001 |
|  |  | Model 2 | 0.983  (0.928; 1.043)  0.576 | 0.973  (0.933; 1.015)  0.201 |  | Model 2 | 0.838  (0.769; 0.913)  <.001 |
|  |  | Model 3 | 0.996  (0.940; 1.055)  0.885 | 0.988  (0.944; 1.034)  0.598 |  | Model 3 | 0.874  (0.787; 0.971)  0.012 |
| Cognitive Impairment |  |  |  |  |  |  |  |
| Date Orientation | $\beta^{b}$  (95% CI)  p-value | Model 0 | 0.017  (-0.029; 0.062)  0.437 | 0.044  (-0.010; 0.099)  0.102 | OR  (95% CI)  p-value | Model 0 | 1.021  (0.951; 1.096)  0.570 |
|  |  | Model 1 | 0.066  (0.021; 0.110)  0.008 | 0.091  (0.028; 0.154)  0.008 |  | Model 1 | 1.062  (0.989; 1.141)  0.098 |
|  |  | Model 2 | 0.046  (0.006; 0.086)  0.027 | 0.076  (0.017; 0.136)  0.017 |  | Model 2 | 1.045  (0.979; 1.114)  0.186 |
|  |  | Model 3 | 0.023  (-0.021; 0.067)  0.278 | 0.049  (-0.010; 0.109)  0.096 |  | Model 3 | 1.034  (0.967; 1.105)  0.326 |

CI, confidence interval; OR, odds ratio; RR, risk ratio; ADL, activities of daily living; IADL, instrumental activities of daily living

^a^ In model 0 we controlled for participant demographics: age, gender, marital status, educational attainment, labor market status, country; socio-economic factors: annual personal income, household net financial assets; health behaviors such as BMI, alcohol consumption, and sports activity; and personality traits: agreeableness, openness, conscientiousness, neuroticism, extraversion. In model 1 we controlled for age and gender, as well as for the prior outcome of interest. In model 2 we controlled for age, gender, labor market status, and socio-economic factors: annual personal income, household net financial assets, as well as for the prior outcome of interest. In model 3, we controlled for all covariates, as in model 0, as well as for the prior outcome of interest. Model 0 (the primary analysis) was adjusted for prior values of the 21 outcome variables, all simultaneously in each regression model. All models were controlled for prior value of the exposure variable.

^b^ All the continuous outcomes were standardized (mean = 0, standard deviation, 1), and β was the standardized effect size.

^c^ The p-value cut-off for Bonferroni correction = 0.05/21 indicators =0.0024)

**Table S6**

Prospective Reciprocal Associations Between Voluntary and/or Charity Work and Emotional Well-Being, Physical Health, Daily Life Functioning, and Cognitive Impairment with Continuous Voluntary and/or Charity Work Variable. Survey of Health, Ageing and Retirement in Europe (SHARE), Adults aged 50 and Over (N=19,821), Unidirectional Associations^a^

| Outcome | Voluntary and/or charity activity 🡪  subsequent PH-EWB-CI-DLF outcome | | PH-EWB-CI-DLF indicator 🡪 subsequent voluntary and/or charity activity | |
| --- | --- | --- | --- | --- |
| Emotional well-being |  |  |  |  |
| Loneliness (3-item loneliness scale) | $\beta^{b}$  (95% CI)  p-value | -0.010  (-0.027; 0.006)  0.207 | $\beta^{b}$  (95% CI)  p-value | -0.012  (-0.027; 0.002)  0.087 |
| Alzheimer’s disease | OR  (95% CI)  p-value | 0.820  (0.708; 0.949)  0.008 | $\beta^{b}$  (95% CI)  p-value | 0.072  (-0.122; 0.266)  0.394 |
| Depression (EURO-D≥4) | RR  (95% CI)  p-value | 1.006  (0.969; 1.004)  0.763 | $\beta^{b}$  (95% CI)  p-value) | -0.038  (-0.077; 0.002)  0.058 |
| Future looks good | $\beta^{b}$  (95% CI)  p-value | 0.027  (0.012; 0.042)  <.001 | $\beta^{b}$  (95% CI)  p-value | 0.028  (0.005; 0.050)  0.019 |
| I feel full of energy these days | $\beta^{b}$  (95% CI)  p-value | 0.037  (0.025; 0.049)  <.001 | $\beta^{b}$  (95% CI)  p-value | 0.036  (0.019; 0.053)  0.001 |
| On balance, I look back on my life with a sense of happiness | $\beta^{b}$  (95% CI)  p-value | 0.027  (0.015; 0.039)  <.001 | $\beta^{b}$  (95% CI)  p-value | 0.011  (-0.003; 0.024)  0.112 |
| I look forward to each day | $\beta^{b}$  (95% CI)  p-value | 0.018  (0.005; 0.031)  0.008 | $\beta^{b}$  (95% CI)  p-value | -0.003  (-0.015; 0.010)  0.646 |
| I feel that my life has meaning | $\beta^{b}$  (95% CI)  p-value | 0.023  (0.004; 0.042)  0.019 | $\beta^{b}$  (95% CI)  p-value | 0.013  (0.003; 0.030)  0.058 |
| Daily life functioning |  |  |  |  |
| ADL  (at least 1 limitation) | OR  (95% CI)  p-value | 0.885  (0.6825; 0.949)  0.001 | $\beta^{b}$  (95% CI)  p-value | -0.044  (-0.118; 0.030)  0.212 |
| IADL  (at least 1 limitation) | RR  (95% CI)  p-value | 0.906  (0.863; 0.951)  <.001 | ($\beta^{b}$  (95% CI)  p-value | -0.029  (-0.083; 0.025)  0.261 |
| Physical health |  |  |  |  |
| Heart attack | RR  (95% CI)  p-value | 0.964  (0.900; 1.032)  0.292 | $\beta^{b}$  (95% CI)  p-value | 0.012  (-0.036; 0.061)  0.569 |
| Hypertension | RR  (95% CI)  p-value | 1.006  (0.954; 1.060)  0.829 | $\beta^{b}$  (95% CI)  p-value | -0.026  (-0.061; -0.008)  0.118 |
| High blood cholesterol | RR  (95% CI)  p-value | 1.001  (0.954; 1.051)  0.954 | $\beta^{b}$  (95% CI)  p-value | -0.054  (-0.095; -0.013)  0.014 |
| Stroke | OR  (95% CI)  p-value | 0.940  (0.846; 1.045)  0.251 | $\beta^{b}$  (95% CI)  p-value | -0.046  (-0.139; 0.046)  0.289 |
| Diabetes | RR  (95% CI)  p-value | 0.966  (0.917; 1.018)  0.197 | $\beta^{b}$  (95% CI)  p-value | 0.003  (-0.051; 0.056)  0.915 |
| Cancer | OR  (95% CI)  p-value | 0.973  (0.894; 1.059)  0.526 | $\beta^{b}$  (95% CI)  p-value | 0.012  (-0.081; 0.106)  0.772 |
| Chronic lung disease | OR  (95% CI)  p-value | 1.052  (0.978; 1.132)  0.174 | $\beta^{b}$  (95% CI)  p-value | -0.099  (-0.168; -0.030)  0.010 |
| Pain | RR  (95% CI)  p-value | 0.952  (0.822; 1.103)  0.515 | $\beta^{b}$  (95% CI)  p-value | -0.036  (-0.074; 0.002)  0.062 |
| Mobility (at least 1 limitation) | RR  (95% CI)  p-value | 0.991  (0.947; 1.037)  0.692 | $\beta^{b}$  (95% CI)  p-value | -0.033  (-0.065; 0.000)  0.050 |
| Cognitive Impairment |  |  |  |  |
| Date Orientation | $\beta^{b}$  (95% CI)  p-value | 0.016  (-0.00; 0.032)  0.052 | $\beta^{b}$  (95% CI)  p-value | 0.002  (-0.016; 0.020)  0.767 |

*Note:* CI, confidence interval; OR, odds ratio; RR, risk ratio; ADL, activities of daily living; IADL, instrumental activities of daily living;

^a^ Missing covariate variables were imputed using chained equations (10 sets of imputed data were generated). All the models were controlled for participant demographics: age, gender, marital status, educational attainment, labor market status, country; socio-economic factors: annual personal income, household net financial assets; health behaviors such as BMI, alcohol consumption, and sports activity; and personality traits: agreeableness, openness, conscientiousness, neuroticism, extraversion. Each model was also adjusted for prior values of the health, daily life functioning and cognitive impairment outcomes and for prior values of the exposure variable. In the model for pain, the prior values of this outcome are not controlled for due to lack of this outcome measured in wave 4. Cross-lagged panel model was estimated. The p-value cut-off for Bonferroni correction = 0.05/21 indicators =0.0024); significant estimates at traditional p<0.05 are bolded.

^b^ All the continuous indicators were standardized (mean = 0, standard deviation, 1), and β was the standardized effect size.

**References in the Supplementary Material**

Ackermann, K. (2019). Predisposed to Volunteer? Personality Traits and Different Forms of Volunteering. *Nonprofit and Voluntary Sector Quarterly*, *48*(6), 1119–1142. https://doi.org/10.1177/0899764019848484

Chan, K. S., Kasper, J. D., Brandt, J., & Pezzin, L. E. (2012). Measurement equivalence in ADL and IADL difficulty across international surveys of aging: Findings from the HRS, SHARE, and ELSA. *Journals of Gerontology - Series B Psychological Sciences and Social Sciences*, *67*(1), 121–132. https://doi.org/10.1093/geronb/gbr133

Dewey, M. E., & Prince, M. J. (2015). Cognitive Function. In A. Börsch-Supan, A. Brugiavini, H. Jurges, J. P. Mackenbach, J. Siegrist, & G. Weber (Eds.), *Health, Ageing and Retirement in Europe. First Results from the Survey of Health, Ageing and Retirement in Europe* (Issues 118–125). https://doi.org/10.1007/978-981-287-080-3_243-1

Guerra, M., Ferri, C., Llibre, J., Prina, A. M., & Prince, M. (2015). Psychometric properties of EURO-D, a geriatric depression scale: A cross-cultural validation study. *BMC Psychiatry*, *15*, 12. https://doi.org/10.1186/s12888-015-0390-4

Hughes, M. E., Waite, L. J., Hawkley Louise, C., & Cacioppo, J. T. (2004). A Short Scale for Measuring Loneliness in Large Surveys: Results From Two Population-Based Studies. *Research on Aging*, *26*(6), 655–672. https://doi.org/10.1109/ICIT.2017.7913066

Jensen, P. H., Lamura, G., & Principi, A. (2014). Volunteering in older age: a conceptual and analytical framework. In A. Principi, P. H. Jensen, & G. Lamura (Eds.), *Active ageing: Voluntary work by older people in Europe* (pp. 21–44). Policy Press.

King, H. R., Jackson, J. J., Morrow-Howell, N., & Oltmanns, T. F. (2015). Personality accounts for the connection between volunteering and health. *Journals of Gerontology - Series B Psychological Sciences and Social Sciences*, *70*(5), 691–697. https://doi.org/10.1093/geronb/gbu012

Mehrbrodt, T., Gruber, S., & Wagner, M. (2019). *Scales and Multi-Item Indicators. SHARE Survey of Health, Ageing and Retirement in Europe*.

Prince, M. J., Reischies, F., Beekman, A. T. F., Fuhrer, R., Jonker, C., Kivela, S. L., Lawlor, B. A., Lobo, A., Magnusson, H., Fichter, M., Van Oyen, H., Roelands, M., Skoog, I., Turrina, C., & Copeland, J. R. M. (1999). Development of the EURO-D scale - A European Union initiative to compare symptoms of depression in 14 European centres. *British Journal of Psychiatry*, *174*(APR.), 330–338. https://doi.org/10.1192/bjp.174.4.330

Rammstedt, B., & John, O. P. (2007). Measuring personality in one minute or less: A 10-item short version of the Big Five Inventory in English and German. *Journal of Research in Personality*, *41*, 203–212. https://doi.org/10.1016/j.jrp.2006.02.001

VanderWeele, T. J. (2017). On the promotion of human flourishing. *Proceedings of the National Academy of Sciences of the United States of America*, *114*(31), 8148–8156. https://doi.org/10.1073/pnas.1702996114
